# Enzyme-Enhanced Manufacturing of Cationized Dialdehyde Cellulose

**DOI:** 10.1021/acs.biomac.4c01819

**Published:** 2025-08-15

**Authors:** Mozhgan Hashemzehi, Helena Håkansson, Gunilla Carlsson Kvarnlöf, Gunnar Henriksson, Björn Sjöstrand

**Affiliations:** † Department of Engineering and Chemical Science, 4209 Karlstad University, Universitetsgatan 2, 65188 Karlstad, Sweden; ‡ Wallenberg Wood Science Center, School of Engineering Sciences in Chemistry, Biotechnology and Health, Royal Institute of Technology, KTH, 100 44 Stockholm, Sweden

## Abstract

In the manufacturing of cellulose derivatives, improving cellulose accessibility is essential for achieving a high product quality. In this study, endoglucanase enzyme treatment was applied prior to the cationization reaction to enhance the accessibility of hydroxyl groups for the production of cationized dialdehyde cellulose (CDAC). A range of enzyme dosages (0.09–45.00 ECU/g) was tested, and their effects on the swelling behavior and surface charge density of the final product were evaluated. The surface charge density of the ultimate cellulosic derivative confirmed its cationization and was proven to enhance the charge density of cationized dialdehyde cellulose (35% increase) compared to untreated pulp with enzyme. Additionally, the modified cellulose exhibited a significantly higher swelling capacity than regular pulps. These findings suggest that enzymatic pretreatment can enhance fiber reactivity and support a more sustainable and efficient production of cellulose-based derivatives, offering a promising potential for commercial applications.

## Graphical abstract

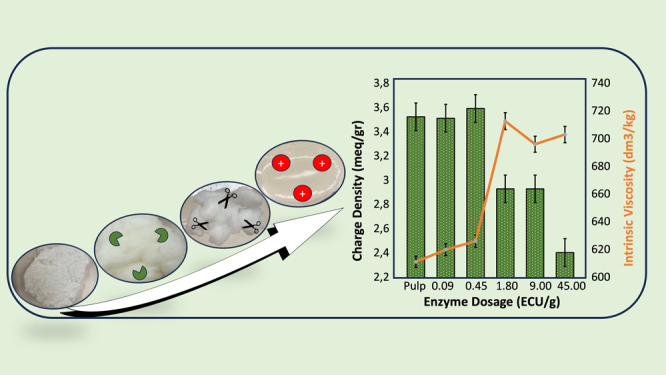

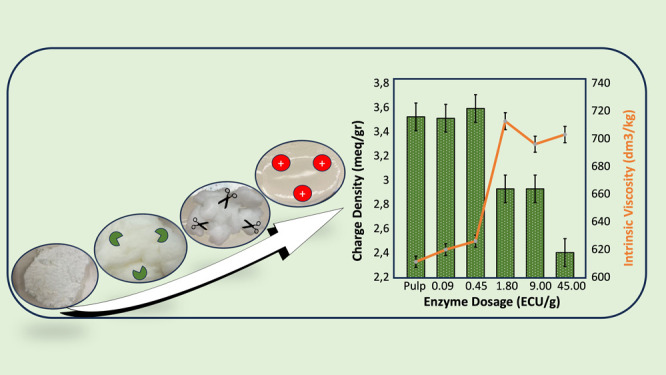

## 1 Introduction

The development of green technologies has become a priority in chemistry, focusing on sustainable solutions to minimize environmental impacts. In this context, deep eutectic solvents (DESs) have gained attention as ecofriendly alternatives to conventional solvents due to their low toxicity, biodegradability, and tunable properties.^71^ A notable application of DESs is their use as a green medium for cellulose modification, particularly for cationic functionalization.^41,44,70^

Cationized cellulose is widely used in various industries, including textiles, pharmaceuticals, cosmetics, food, and papermaking, due to its biodegradability, cost-effectiveness, and low toxicity.^20,70^ In the pulp and paper industry, cationized cellulose has been introduced as a replacement for conventional cationic polymers, improving fiber bonding and mechanical properties.^17,21^ The effectiveness of cationized cellulose as an additive depends largely on its charge density, which influences its swelling behavior, flexibility, and binding efficiency.^21,43^ Enhancing the charge density of cationized cellulose requires increasing the accessibility of hydroxyl groups, allowing for improved interactions between reactants and cellulose fibers.^40^

To enhance the accessibility to hydroxyl groups or reactivity of cellulose fibers, different methods have been introduced for example, mechanical treatment,^17,38,39^ chemical treatment,^38,67,72^ ultrasonic,^38,47^ thermal degradation treatment,^11,38^ and enzymatic methods.^6,15,38,59^ Among these, enzymatic treatments have emerged as an environmentally friendly, efficient, and highly selective method for modifying cellulose structure while maintaining its integrity.^36,69^

However, cellulose’s crystalline and resistant structure makes it a challenging substrate for degradation. In nature, aerobic fungi cellulases work cooperatively to degrade cellulose,^3^ including key enzymes such as cellobiohydrolases (EC3.2.1.91), and endoglucanases (EC3.2.1.4).^54^ Endoglucanases play a crucial role by randomly cleaving internal bonds within the cellulose chains, creating new chain ends that serve as substrates for cellobiohydrolases.^26^

While cellobiohydrolases degrade cellulose from the ends, endoglucanases cleave randomly within the chains, targeting regions with disrupted structures, thus enhancing cellulose reactivity.^28,69^ This internal cleavage of β-1,4-glycosidic bonds reduces the degree of polymerization (DP), breaking cellulose into shorter chains. As a result, the solubility of cellulose increases, making it more accessible for further processing and modification.^25^

Cao and Tan studied the effect of different enzymes, including multicomponent cellulases, purified endoglucanases, and cellobiohydrolases, on the DP reduction rate. Their finding showed that endoglocanase is particularly efficient in hydrolyzing pulp, reducing the intrinsic viscosity and DP, and increasing the alkaline solubility of the substrate. In contrast, cellobiohydrolase had a less noticeable impact on these properties.^7^

Several hypotheses have been proposed regarding the mechanism of pulp activation by endoglucanases. One such hypothesis suggests that endoglucanases target less ordered cellulose regions, leading to fiber wall swelling,^4,29,37^ which enhances cellulose accessibility to solvents and reactants for further reactions.^15^ This swelling effect is crucial for the modification of cellulose’s structural properties, enhancing its reactivity and making it more suitable for industrial processes.^26^ Chiriac et al. (2014) demonstrated the potential of endoglucanases to improve cellulose reactivity, showing that dissolving cellulose in a low-temperature NaOH solution was more effective when endoglucanases were applied.^12^ This enhanced reactivity can be attributed to the swelling of the fiber, where internal stresses disrupt the cellulose structure and break some intermolecular bonds. As a result, the degree of crystallinity decreases, and the surface area of the fiber increases, making it more accessible for further processing.^45^

Therefore, endoglucanases are particularly valuable in various technical applications. For instance, pure fungal endoglucanases are highly efficient in reducing energy consumption during nanocellulose production,^24,58^ and enhancing the chemical reactivity of dissolving pulps in the viscose process.^10,23,58^

While endoglucanases can enhance fiber swelling and accessibility, the effectiveness of enzymatic treatments is influenced by several key factors, such as enzyme concentration, reaction time, temperature, and pH.^30^ Controlled enzymatic treatment can improve hydroxyl group accessibility and fiber porosity, but excessive swelling may lead to fiber degradation, increased fines content, and a decrease in pulp properties.^61^ Therefore, optimizing the enzyme dosage is crucial to achieving the desired modifications while maintaining fiber integrity.

While the combination of periodate oxidation and Schiff base reaction by using deep eutectic solvent has been previously studied in cationization of cellulose,^21,41^ the combination of three steps, enzymatic treatment, periodate oxidation, and Schiff base reaction, remains underexplored. In this work, we have investigated the potential of utilizing a commercial endoglucanase as a pretreatment step, followed by periodate oxidation to generate dialdehyde cellulose and subsequent reaction to introduce cationic groups, utilizing a deep eutectic solvent (DES). The study specifically has examined the impact of varying enzyme dosages (0.09, 0.45, 1.80, 9.00, and 45.00 ECU/g) during the pretreatment step on the swelling behavior and charge density of the final cationized dialdehyde cellulose. By evaluating the synergy of these treatments, this work provides new insight into how enzyme-induced structural changes influence downstream reactivity, leading to the development of highly charged cationic dialdehyde cellulose (CDAC) suitable for papermaking and related applications.

## 2 Materials and Methods

### 2.1 Materials

Northern beaten bleached softwood kraft pulp was used as the starting material for the cellulose-based cationized derivatives. A monocomponent endoglucanase (ECOPULP L900) as an effective enzyme for reducing the viscosity and increasing the swelling of pulps was manufactured and supplied by AB Enzymes. The enzyme used in this study was produced by using a genetically modified strain of *Trichoderma reesei*. The cellulase activity was determined by the manufacturer and expressed in endocellulase units (ECU) per gram of material as 90000 ECU/g. Lithium chloride (99%) and sodium periodate (>99%) were used for periodate oxidation of the pulp into dialdehyde cellulose (DAC). Ethanol (96%), glycerol, and aminoguanidine hydrochloride (>98%) were used for the cationization of DAC. Sodium polyethylene sulfonate (PES-Na) and polydiallyldimethylammonium chloride (polyDADMAC) were used as a polyelectrolyte to determine the cationic charge by back-titration. Deionized water and ethanol were used for washing. All reagents were supplied by VWR (Sweden).

### 2.2 Method

#### 2.2.1 Enzyme Treatment

In this study, the synthesis of cationized dialdehyde cellulose (CDAC) was carried out through a three-step procedure. First, the softwood pulp underwent an adjustment to pH 7 by using a phosphate buffer composed of NaH_2_PO_4_ and Na_2_HPO_4_. Then, 0, 0.09, 0.45, 1.80, 9.00, and 45.00 ECU/g dosage of enzyme was added. To ensure an even distribution of the enzyme, they were added to the buffer before adding the buffer into the pulp, resulting in a final pulp concentration of 3%. The enzymatic incubation was conducted in sealed plastic bags submerged in a water bath set at 50 °C, with each pulp sample weighing 30 g in dry weight. At regular intervals, the samples were removed from the bath and briefly kneaded for 10–15 s to ensure uniform mixing. After the incubation period, the samples were washed on a Buchner funnel using deionized water at 80 °C and then transferred to an 80 °C water bath for 30 min to deactivate the enzyme. The treated pulp was subsequently filtered using a Buchner funnel and rinsed with 1000 mL of deionized water.

#### 2.2.2 Periodate Oxidation

Moving on to the second step, the synthesis of DAC, 10 g of enzyme treated pulp was diluted and heated to a final temperature of 70–75 °C. Then, 18 g of lithium chloride (LiCl) and 8.2 g of sodium periodate (NaIO_4_) were added and left to react for 3 h. Given the light sensitivity of NaIO_4_, this reaction was performed under dark conditions. The DAC fibers were washed with 96% ethanol and water.^21,41^

#### 2.2.3 Cationization

Finally, the cationization of DAC was carried out using deep eutectic solvents. First, deep eutectic solvents as both reaction medium and reagent were formed for the production of CDAC. Aminoguandine hydrochloride and glycerol were mixed under 90 °C conditions for 30 min until a clear solvent (DES) was formed. Then, DAC from the previous stage were added to the DES solvent at 75 °C, and the reaction was stirred for 30 min. Finally, CDAC was washed thoroughly with ethanol. Figure 1 shows that the process consisted of enzyme treatment and then producing dialdehyde cellulose by selectively oxidizing the C_2_–C_3_ groups of the glucose ring to form two aldehyde groups. These aldehyde groups were then reacted with DES and resulting in a positive charge to the cellulose.^21,41^

**Figure 1:**
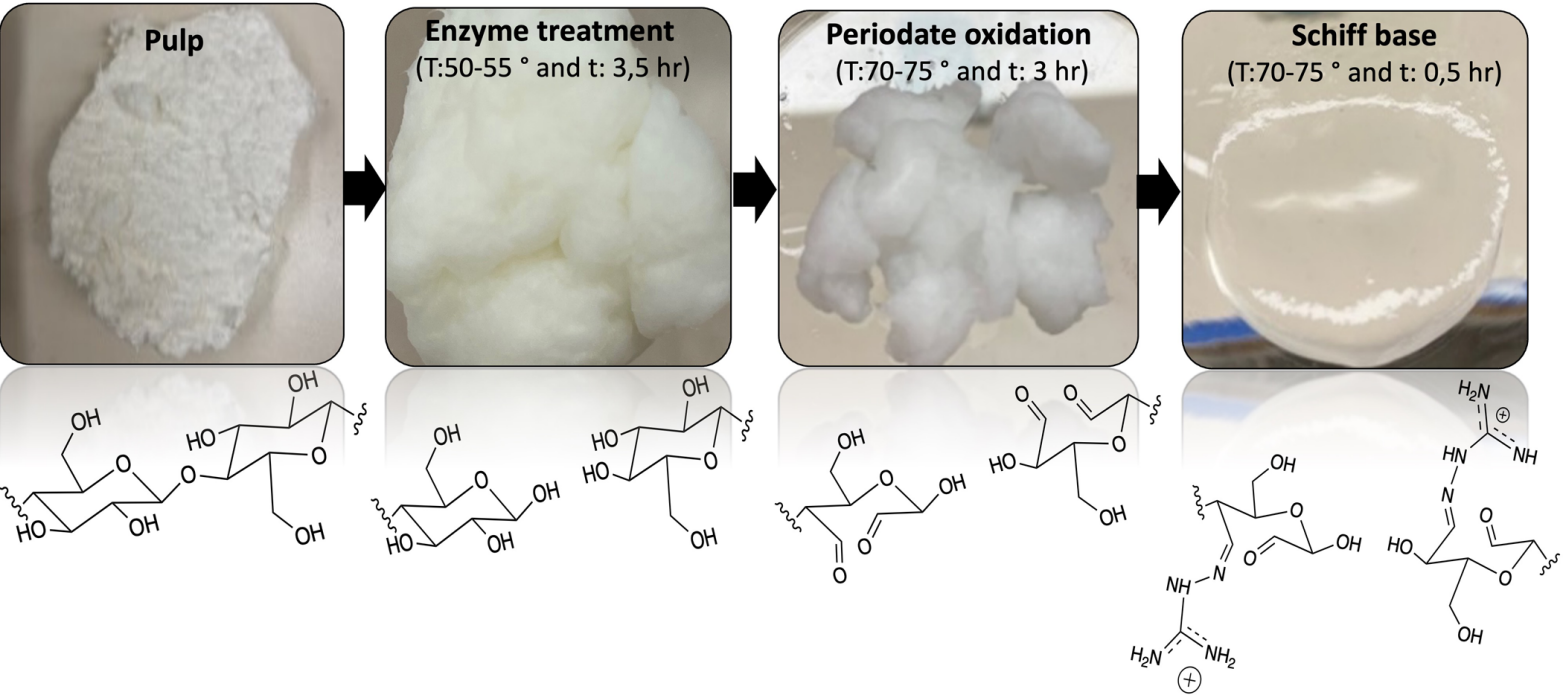
Mechanism of cationization of cellulose in this study.

## 3 Characterization

### 3.1 Fourier Transform Infrared Spectroscopy (FTIR) Analysis

Fourier transform infrared (FTIR) analysis of both DAC and CDAC samples was conducted by using a Cary 630 FTIR spectrometer from Agilent Technologies. The spectral data were collected within a wavenumber range of 700 cm^–1^ to 4,000 cm^–1^. The structural properties of cellulose can be studied using FTIR analysis by comparing the intensity of a functional group peak.^48^ The lateral order index (LOI) is calculated as the ratio of the band at 1430 cm^–1^ to 897 cm^–1^. The total crystallinity index (TCI) was also determined by comparing the ratio of 1372 cm^–1^ (C–H bending) to 2900 cm^–1^ (C–H and CH_2_ stretching). Finally, the hydrogen bonding index (HBI) was obtained by comparing the intensity ratio of the 3340 cm^–1^ and the 1330 cm^–1^.^19^

### 3.2 Intrinsic Viscosity and Depolymerization Measurement

The intrinsic viscosity of the pulps was measured according to the ISO 5351:2010 standard method. The intrinsic viscosity number gives an average value of the length of cellulose chains, and the degree of polymerization can be calculated from it. Several correlations between the molecular weight and the intrinsic viscosity have been suggested,^31,49^ but in this case when we compared the depolymerization of similar samples no recalculations were made.

### 3.3 Degree of Oxidation of DAC

The aldehyde content was measured by titration. 0.1 g of fibers was mixed with 25 mL of 0.25 M hydroxylamine hydrochloride and stirred for 2 h. After that, the fibers were filtered, and the filtrate was titrated back to pH 4 using 0.1 M sodium hydroxide. The degree of oxidation was calculated based on the amount of sodium hydroxide used.^76^

### 3.4 Particle Charge Density Measurement

For determining the cationic charge in cellulose derivatives, particle charge density (PCD) measurement was considered the most reliable method because it removes impurities and side reactions.^64^ This method indirectly determines the fiber charge by means of a reverse titration process. The fibers were submerged in a polyelectrolyte with an opposing charge. This mixture was stirred for approximately 2 h using a magnetic stirrer. Throughout this duration, the anionic polyelectrolyte, polyethylene sodium sulfonate (PESNa), neutralized all of the cationic charges present in the sample. As an excess quantity of PESNa was used, a residual anionic charge remained within the sample. This polyelectrolyte, after coming into contact with the cationic fiber, was titrated using a cationic polyelectrolyte known as poly­(diallyldimethylammonium chloride) or poly-DADMAC (V1). A similar titration was carried out for the polyelectrolyte before it came into contact with the fiber (V2). The variation in charge indicates the quantity of polyelectrolyte taken up by the fibers, thereby disclosing their charge density.

$$q=\frac{\left(V2-V1\right)\times C}{m}$$

- *m*: Solid content of mixture
- *C*: Titrant concentration

### 3.5 Polarized-Light Optical Microscopy

The reference pulp and CDAC treated with different dosage of enzyme was examined using a polarized-light optical microscope (Olympus BX51, Tokyo, Japan). This microscope is outfitted with two polarizers. Polarized light can improve the detection of swelled fibers by improving contrast and the quality of the image. The employment of polarized light enhances the identification of expanded fibers by enhancing both the contrast and image quality. The specimens were observed at a 10×/0.25 magnification under the microscope.

### 3.6 Water Retention Value (WRV)

The WRV of the treated samples was measured according to the standard of ISO 23714 (2014). The approximately 20% dry samples were diluted with deionized water to 5–10 g per liter, and centrifuged during 30 min, 3000*g* controlled to 23 °C. Differences from the standard were the higher concentration and the skipped disintegration prior to centrifugation; this was to ensure that enough material stayed in the sample holder and did not escape through the mesh. The centrifuged sample was weighted before and after drying overnight in an oven at 105 ± 0.5 °C. The WRV was then calculated by eq 1, and a 95% confidence interval was calculated to show how the method varied between the two tests of each, according to the standard.

$$WRV\;\left(\frac{g}{g}\right)=\frac{wet\;sample\;\left(g\right)}{dry\;sample\;\left(g\right)}-1$$

## 4 Results and Discussion

### 4.1 FTIR Spectroscopy

This study assessed the effectiveness of enzymatic treatment (cellulase of endoglucanase type) on pulps using varying doses of endoglucanase. Figure 2 illustrates the chemical changes in fibers after enzymatic treatment (using a cellulase of the endoglucanase type), periodate oxidation, and cationization. All the curves exhibit the typical bands of cellulose at 3330, 2901, 1429, 1362, 1156, 1024, and 894 cm^–1^.^65^

**Figure 2:**
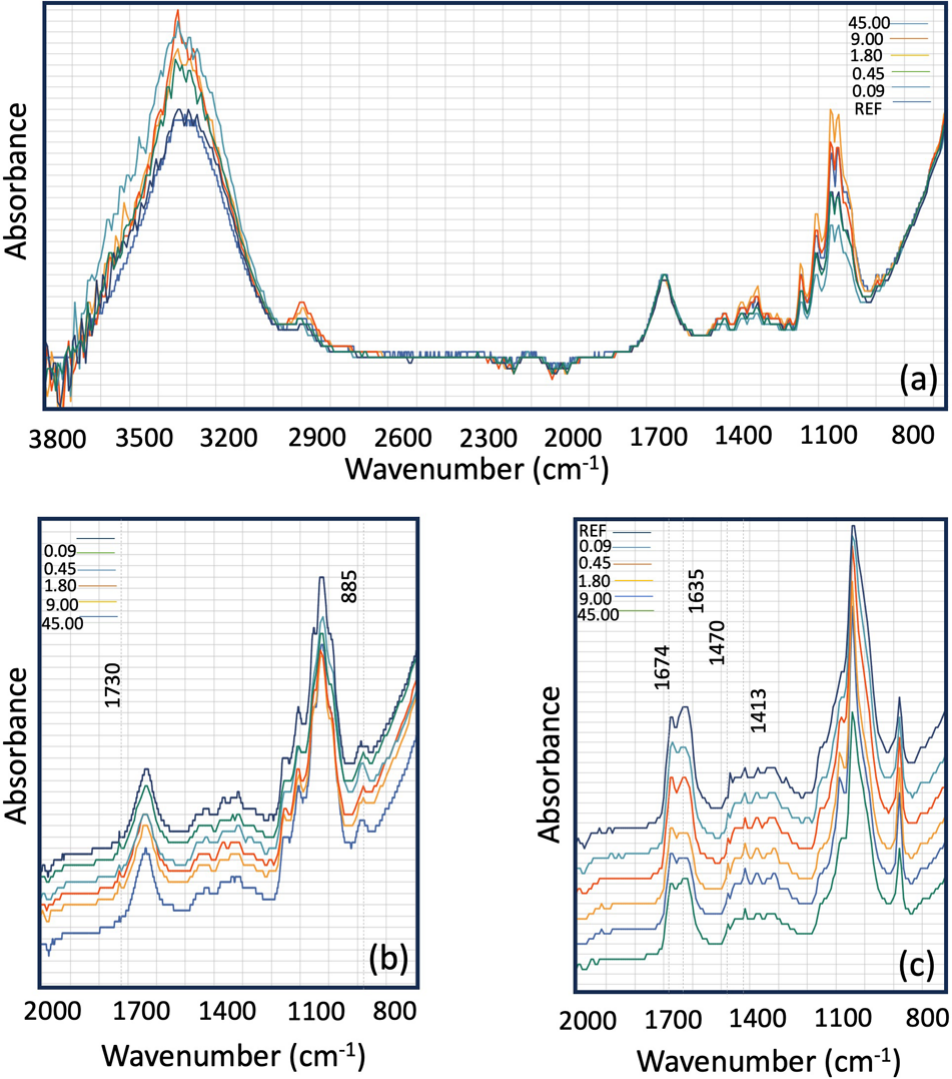
FTIR spectra of samples after (a) enzyme treatment (EN), (b) periodate oxidation (DAC) and (c) Schiff base reaction (CDAC) when 0, 0.09, 0.45, 1.80, 9.00, and 45.00 ECU/g dosage of enzyme was added in the first stage of pretreatment.

In Figure 2(a), the band near 1160 cm^–1^ corresponds to the antisymmetric stretching of C–O–C bonds, characteristic of cellulose and hemicellulose structures. Similarly, the band around 1318 cm^–1^ is attributed to CH_2_ wagging vibrations, also associated with cellulose and hemicellulose.^7^ An indicator for assessing the effects of endoglucanase treatment is the amorphous region of cellulose, identified by the absorption band at 897 cm^–1^.^49^ The bands around 3400 cm^–1^ correspond to the OH vibrations. This band was broad and shifted to a lower wavenumber (3370 cm^–1^) due to the presence of intermolecular and intramolecular hydrogen bonds. After enzymatic hydrolysis, the OH vibration bands became narrower and shifted back to a higher wavenumber (3400 cm^–1^). This suggests that a portion of the hydrogen bonds were disrupted during the enzymatic hydrolysis process.^8,63^

In Figure 2(b), the FT-IR spectrum of DAC exhibits characteristic bands at 1,730 cm^–1^, corresponding to aldehyde carbonyl groups, and at 885 cm^–1^, which are associated with hemiacetal bonds formed between aldehyde groups and neighboring hydroxyl groups^2,65^ remains visible. These features demonstrate the successful introduction of the aldehyde or hemiacetal groups onto the surface by sodium periodate oxidation. In Figure 2(C), which shows the FTIR spectra of CDAC, several new bands are observed at 1674 cm^–1^ and 1635 cm^–1^, corresponding to the carbon–nitrogen double bond of imines and nitrogen–hydrogen bond bending, respectively.^41^ These results suggest that CDAC was formed under the specified conditions. However, distinguishing variations in peak intensity across samples treated with different enzyme dosages can be challenging. To ensure a more accurate interpretation of the structural changes, we complemented the FTIR analysis with additional techniques. These included crystallinity indices (LOI, TCI, HBI), intrinsic viscosity measurements to monitor changes in polymer chain length, and water retention value (WRV) measurements to evaluate fiber swelling and crystallinity effects.

Crystallinity indices, such as LOI, TCI, and HBI, were calculated from FTIR spectra to assess structural modifications in cellulose. These indices provide a qualitative and relative evaluations of crystallinity insight into the relative proportions of crystalline versus amorphous domains, which are affected by enzymatic and chemical treatments.^55^ This was done by comparing the intensity of a functional group peak linked to the crystalline region with that of another functional peak representing the amorphous region in each FTIR spectrum.^50^ The crystallinity indices (LOI, TCI, and HBI) of enzyme-treated CDAC samples are shown in Table 1.

**Table 1:** Crystallinity Indices before and after Enzyme Treatment and Schiff Base Reaction^a^

sample	LOI (1430/897 cm^–1^)	TCI (1370/2900 cm^–1^)	HBI (3340/1330 cm^–1^)
pulp	0.635	1.251	4.087
En-0.09	0.602	1.136	4.370
En-0.45	0.614	0.928	5.238
En-1.80	0.550	1.159	6.190
En-9.00	0.561	1.070	5.715
En-45.00	0.556	1.083	5.500
CDAC	0.615	0.738	4.135
CDAC-0.09	0.602	0.939	4.622
CDAC-0.45	0.613	0.929	5.081
CDAC-1.80	0.548	0.831	7.112
CDAC-9.00	0.523	0.919	5.468
CDAC-45.00	0.523	0.934	5.500

a LOI: lateral order index or crystallinity index based on FTIR. TCI: total crystallinity index based on FTIR. HBI: hydrogen bonding index based on FTIR.

The lateral order index (LOI) is determined as the ratio of the absorption band at 1430 cm^–1^ to that at 897 cm^–1^ in FTIR spectroscopy. The band at 1430 cm^–1^, attributed to CH_2_ scissoring vibrations, is strong in cellulose I but weak in cellulose II and amorphous cellulose. Nevertheless, the absorption band at 897 cm^–1^, associated with C–O–C stretching at the β-(1,4)-glycosidic linkage, is weak and broad in cellulose I but sharp and strong in cellulose II and amorphous cellulose.^51^ The total crystallinity index (TCI) is calculated by comparing the ratio at 1372 cm^–1^ (C–H bending) to 2900 cm^–1^ (C–H and CH_2_ stretching) and is used to evaluate the crystallinity of cellulose materials.^50,56^

Table 1 demonstrates a slight reduction in both TCI and LOI values following enzymatic pretreatment. Specifically, the LOI decreased from 0.635 to 0.55, while the TCI declined from 1.251 to 0.928. Untreated fibers exhibited the highest TCI and LOI values, reflecting a higher degree of crystallinity and a more organized cellulose I structure compared with enzyme-treated samples. These observed decreases in TCI and LOI suggest that enzymatic pretreatment disrupts the crystalline regions within the cellulose structure. The hydrogen bonding index (HBI) is calculated by comparing the intensity ratio of two specific FTIR peaks: the 3340 cm^–1^ peak, which corresponds to νOH stretching vibrations caused by intra- and intermolecular hydrogen bonds, and the 1330 cm^–1^ peak, which is associated with δOH bending vibrations of hydroxyl groups at O_2_H and O_3_H. HBI serves as a qualitative measure of cellulose crystallinity; an increase in HBI generally corresponds to a decrease in crystallinity.^33,52,73^ Therefore, the increase in HBI from 4.087 to 6,190 in enzyme treated samples may indicate a reduction in overall crystallinity.

CDAC derivatization led to a further reduction in the LOI and TCI values, highlighting the impact of chemical modifications on the cellulose structure. This trend suggests more crystalline disruption, likely due to the incorporation of quaternary ammonium groups by deep eutectic solvents. In contrast, HBI values increased following CDAC derivatization with CDAC-1.8 reaching 7.112. Together, these findings indicate that CDAC derivatization promotes a shift toward a more amorphous cellulose structure, enhancing its reactivity and functional potential. These qualitative and relative assessments of crystallinity^75^ can be further supported by water retention value (WRV) measurements. WRV provides important information about the swelling behavior of cellulose, which is closely linked to its degree of crystallinity.^53^ Since swelling primarily occurs in the amorphous regions, greater swelling generally indicates a lower crystallinity.

### 4.2 Effect of Enzyme Treatment on Intrinsic Viscosity

The effectiveness of the enzymatic treatment on pulp was determined by assessing the reduction in intrinsic viscosity, which reflects the average degree of polymerization of cellulose. A decrease in viscosity indicates chain cleavage, particularly in the amorphous regions, which can enhance reactivity by generating more accessible sites.^14,25^ As shown in Figure 3, the pulp intrinsic viscosity decreased from 716 to 618 dm^3^/kg with the highest enzyme dosage, with the most significant drop occurring at 1.8 ECU/g. This reduction can be attributed to the action of endoglucanases (EG), which primarily cleave internal β-1,4-glycosidic linkages in cellulose, leading to a decrease in polymer chain length and thus lowering the intrinsic viscosity.^26^

**Figure 3:**
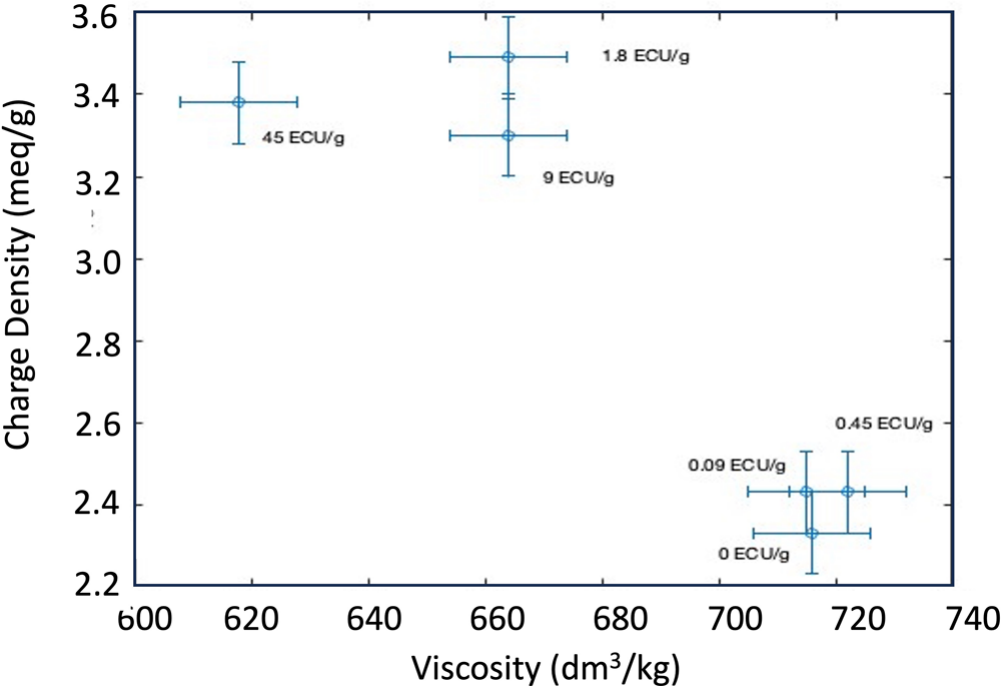
Correlation of intrinsic viscosity and charge density of pulp when 0, 0.09, 0.45, 1.80, 9,00 and 45,00 ECU/g dosage of enzyme has been added.

However, when enzyme concentrations were increased further, no significant reduction in the intrinsic viscosity was observed. Previous research by Klemm et al. (2011) and Hidayat et al. (2012) suggested that since endoglucanases mainly target disordered regions along the fibers, these limited sites may become saturated, thereby preventing further enzyme activity at higher dosages.^22,32^

### 4.3 Swelling behavior of Enzyme-Pretreated Fibers

To support the relative crystallinity assessments presented in Table 1, water retention value (WRV) measurements were conducted to evaluate the impact of enzymatic treatment on fiber swelling behavior, which is indicative of accessibility changes in the cellulose structure.^9^ Increased WRV reflects enhanced water uptake capacity, often associated with disruption of crystalline regions and loosening of the fiber wall.^46^ Figure 4 shows that the WRV increased after the first step, enzyme treatment. As the enzyme dosage increased, more amorphous or less ordered regions appeared on the surface and between microfibrils. This structural change was reflected in the swelling behavior of the cellulose, which grew by less than 10%. At a low enzyme dosage (0.45 ECU/g), fiber swelling increased slightly, resulting in minimal change in WRV. This is due to the fact that endoglucanase primarily targets disordered cellulose regions between and on the surface of fibrils, as well as shorter segments within fibril aggregates. This enzymatic action disrupts the fiber structure, promoting fiber wall swelling and improving accessibility.^24^ As the enzyme dosage increased to 1.80 ECU/g, fiber accessibility and swelling were optimally enhanced, leading to a more significant increase in WRV. However, further increasing the dosage to 9.00 ECU/g did not yield additional WRV improvement, likely due to enzyme saturation and a jamming effect that restricts enzyme activity.

**Figure 4:**
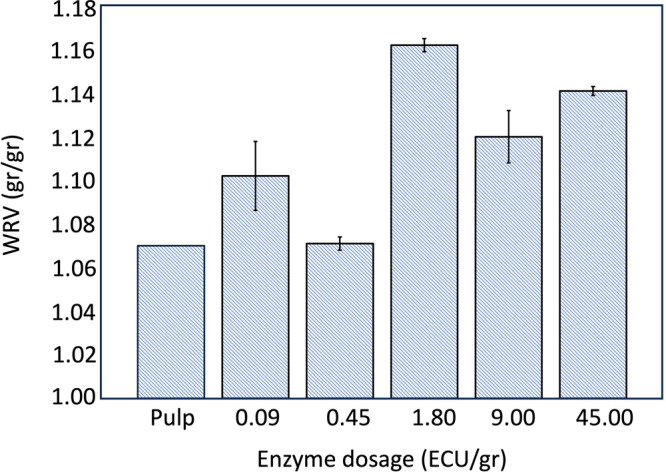
WRV of pulp after enzyme treatment; 0, 0.09, 0.45, 1.80, 9.00, and 45.00 ECU/g dosage of enzyme was added. Error bars indicate 95% confidence intervals based on two repetitions.

### 4.4 Degree of Oxidation in Enzyme-Pretreated DAC

Although enzymatic pretreatment can improve fiber accessibility, it is not sufficient on its own to significantly enhance the efficiency of the Schiff base reaction due to the lack of reactive aldehyde groups. This limitation became apparent when samples that were treated only with the enzyme, without undergoing the intermediate periodate oxidation step, were subjected directly to cationization. These samples showed very low charge densities (∼0.01–0.02 mequiv/g), which is likely due to the presence of only a small fraction of aldehyde groups at the cellulose chain ends. These groups exist in equilibrium with cyclic hemiacetals and account for limited reducing functionality.^64^ These findings highlight the vital role of reactive aldehyde groups in enabling effective cationization. To enhance the reactivity, periodate oxidation was employed to introduce additional aldehyde functional groups and simultaneously alter the cellulose structure. It is important to note that the reaction conditions for aldehyde formation are sensitive and must be carefully controlled. Side reactions, such as the formation of intramolecular hemiacetals between aldehyde and hydroxyl groups, can reduce the number of reactive sites. These equilibria complicate aldehyde quantification and may reduce the overall reactivity in subsequent functionalization steps. Therefore, while periodate oxidation is applied to increase the reactive sites, its efficiency is influenced by factors such as oxidant concentration, reaction time, and pH, which must be optimized to minimize side reactions and maximize functional aldehyde availability.^62^

In periodate oxidation, the objective is to cleave the C2–C3 bonds in the glucose units of cellulose, thereby generating dialdehyde functionalities. This cleavage disrupts the crystalline structure and exposes additional reactive sites, enhancing the material’s reactivity. The efficiency of this oxidation process was evaluated by quantifying the aldehyde content of the resulting dialdehyde cellulose (DAC), using titration to determine the degree of oxidation. This parameter is critical for assessing the success of the aldehyde group introduction, which directly impacts the potential for subsequent Schiff base formation.

After 3 h of periodate oxidation treatment, the aldehyde content increased by 45% in enzyme-pretreated samples (Figure 5). This improvement is attributed to the enzyme disrupting the hydrogen bonding network within cellulose, which leads to greater exposure of hydroxyl groups to the oxidizing agent. However, at enzyme concentrations above 1.8 ECU/g, the oxidation efficiency declined. This phenomenon can be attributed to enzyme crowding and surface saturation, as outlined by Igarashi et al. (2011). Their study demonstrated that at high enzyme concentrations, cellulases begin to compete for limited binding sites on the cellulose surface. This competition leads to crowding or jamming, where enzymes obstruct each other’s access and movement, thereby reducing hydrolysis efficiency.^27^ In our case, this crowding likely limited the exposure of hydroxyl groups to the oxidizing agent, diminishing the effectiveness of periodate oxidation at higher enzyme dosages.^5,66^

**Figure 5:**
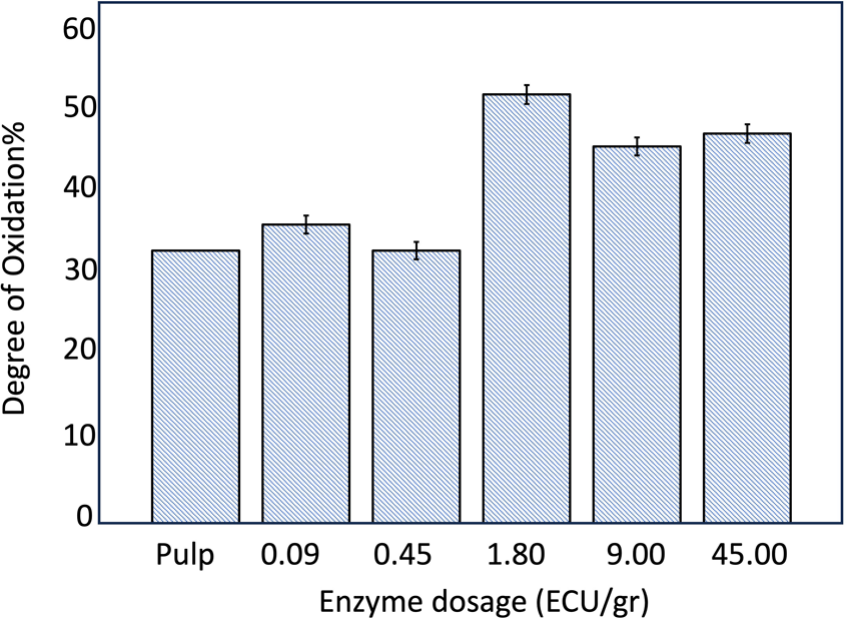
Degree of oxidation of DACs when 0, 0.09, 0.45, 1.80, 9.00, and 45.00 ECU/g dosage of enzyme was added. Error bars indicate 95% confidence intervals based on three repetitions.

### 4.5 Charge Density of Enzyme-Pretreated CDAC

By cleaving C2–C3 bonds and converting secondary hydroxyl groups into reactive aldehyde groups, periodate oxidation enhances the reactivity of pulp, making it more suitable for further functionalization.^63^ In the next step, DACs underwent Schiff base reactions using deep eutectic solvents, where cationization occurred through interactions between aldehyde groups and the DES solvent. Functionalization occurs via amination using 1,3-diaminoguanidine hydrochloride in DES medium. In this system, the DES is composed of glycerol and 1,3-diaminoguanidine hydrochloride. As glycerol acts as the hydrogen bond donor through its hydroxyl groups, the amino groups of 1,3-diaminoguanidine hydrochloride serve as hydrogen bond acceptors based on prior studies.^41^

This DES demonstrated a strong capability to disrupt the hydrogen bonding network within cellulose. It has been shown that the prepared DES can act as ‘molecular scissors,’ effectively breaking the intermolecular hydrogen bonds and altering the structural integrity of cellulose.^74^ The reaction between aldehyde groups on the cellulose and amino groups from the Schiff base led to the grafting of guanidine salts onto the cellulose, thereby imparting a positive charge to the cellulose. The extent of amination was closely linked to the charge density; greater grafting resulted in a higher cationic charge density. Figure 8 presents the charge density of CDAC after a 30 min reaction. Charge densities ranged from 2.3 to 3.6 mequiv/g, with an increase observed between enzyme dosages of 0.45 and 1.80 ECU/g. This increase is directly linked to the higher enzyme dosage used during the pretreatment step. The elevated charge density of the cationized cellulose serves as an indicator of enhanced pulp reactivity achieved through the three-step treatment process.

Previous studies by Duan et al. (2016), Gehmayr (2012), and Köpcke (2010) have shown that enzymatic pretreatment improves pulp reactivity for downstream chemical modifications.^13,18,34,35^ Since endoglucanase (EG) contribute not only to the reduction of cellulose polymer chain length but also to the enhancement of fiber porosity and the microfibril distance in fibers, suggesting that these enzymes may help disrupt the structural cohesion of microfibril bundles within the fiber walls.^14,15,17^ These changes are directly linked to fiber accessibility, which plays a crucial role in determining the reactivity of cellulose.^42^ Increased fiber accessibility facilitates the more effective penetration of chemicals, thereby enabling more thorough and efficient chemical interactions with the cellulose structure.^16^ Rahikainen et al. (2019) also reported that enzymatic treatment promotes the formation of a more porous fiber wall structure, which is beneficial for increasing pulp reactivity during chemical modification.^57^ Thus, enzymatic pretreatment not only contributes directly to fiber activation but also synergistically amplifies the effectiveness of the subsequent oxidation and cationization steps.

### 4.6 Observing Microstructure of Samples by Optical Microscopy

Polarized light microscopy was employed to examine the effects of different enzyme dosages on the structural changes in pulp fibers. Under polarized light microscopy, pulp samples treated with high enzyme dosages looked very similar to those treated with low dosages. However, differences were observed when comparing the various steps of treatment, enzyme treatment, periodate oxidation, and Schiff base reaction. After enzyme treatment, the structure appeared dense and tightly interwoven. It is difficult to reveal signs of enzyme-induced degradation by optical microscopy. Following periodate oxidation (Figure 6(d), (e), and (f)), the surface became smoother, with fibers appearing thinner. Cationized samples, Figure 6(g), (h) and (i), displayed swelling behavior. The swelling process did not uniformly impact the entire fiber; rather, the darker central core (lumen) remained visible, while the lighter outer indicated where expansion had occurred. It is clear that Figure 6(g) shows a structure change in fibers, while Figure 6(i) displays more changes and swelling. It should be noted that that optical microscopy with polarized light provides a qualitative approach to observe structural changes resulting from enzymatic treatments. However, this method alone may not provide conclusive insights into swelling behavior. So, we characterized samples by the water retention value method which reveals more precise information about fiber swelling behavior.

**Figure 6:**
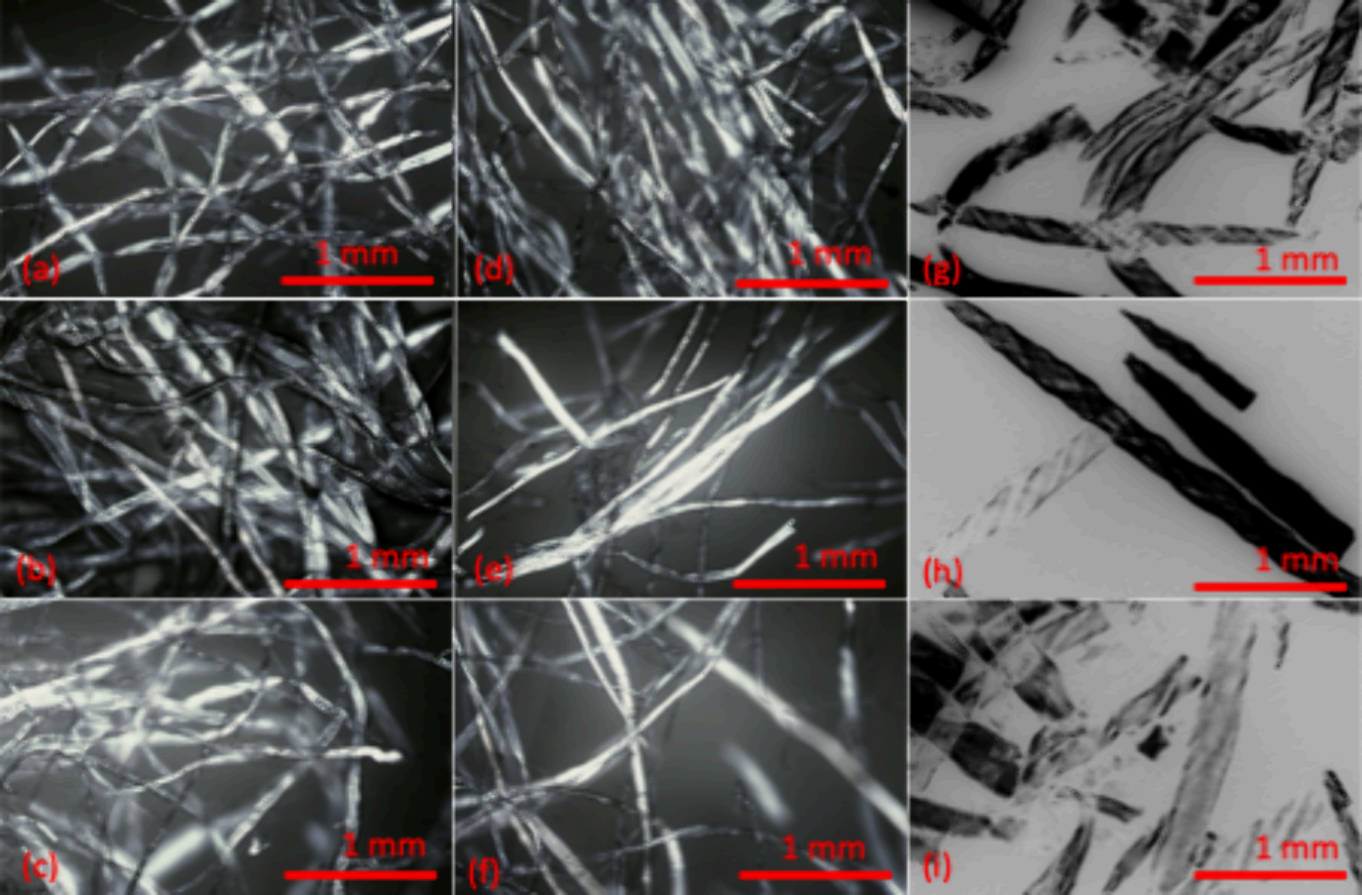
Polarized light optical microscopy images of the pulp samples with enzyme treatment of different dosages: a) Pulp pretreated with 0.09, c) pulp pretreated with 1.80 ECU/g, d) DAC, e) DAC when pulp pretreated with 0.09 ECU/g, f) DAC when pulp pretreated with 1.80 ECU/g, g) CDAC, h) CDAC when pulp pretreated with 0.09 ECU/g, i) CDAC when pulp pretreated with 1.80 ECU/g.

### 4.7 Swelling behavior of Enzyme-Pretreated CDAC

Water retention values of CDACs (Figure 7) show that the swelling is extremely high compared to an untreated sample of the same pulp^60^ which showed a WRV of 1.4 g/g. Note that the materials were difficult to retain in the sample holder during centrifugation, with a certain amount of material leaking through the mesh. The sample treated with 1.80 ECU/g enzyme dosage resulted in too swollen material to retain in the WRV sample cup, leaving insufficient material for accurate weighting. The results (Figure 7) show that fiber swelling reached its peak at moderate enzyme dosages, where the combined effect of enzymatic and chemical treatments appears to be most effective. This trend correlates with the observed increase in charge density and the decrease in intrinsic viscosity at midrange enzyme additions (Figure 8). The lack of significant swelling at high enzyme concentrations may be attributed to the jamming effect. Since enzyme movement on cellulose fibers is restricted to one dimension along the fiber’s surface. When enzyme concentrations are high, enzyme molecules aggregate on a limited cellulose surface area, hindering each other’s progress. As a result, enzymatic activity become less efficient and does not increase further.^5,68^

**Figure 7:**
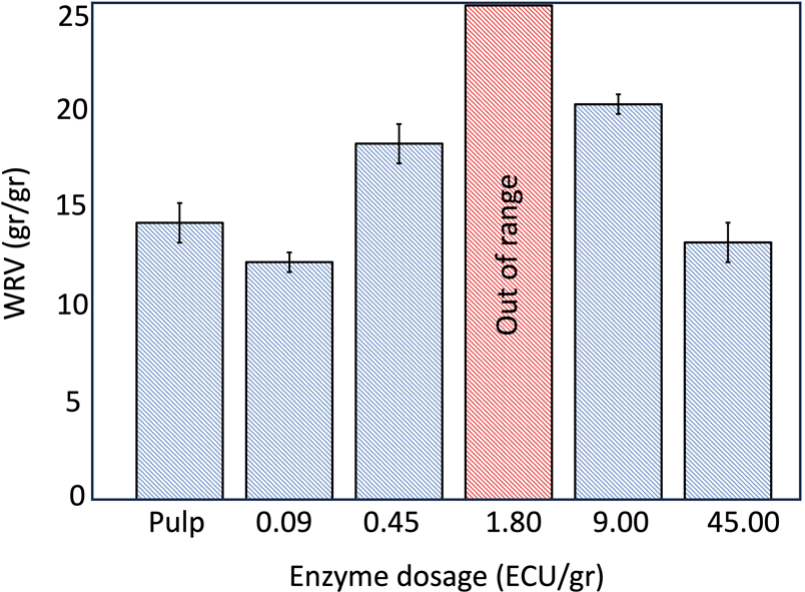
WRV (g/g) of cationized dialdehyde cellulose following enzyme treatment, periodate oxidation, and Schiff base reaction. Error bars indicating 95% confidence intervals based on two repetitions. Note that 1.80 ECU/g dosage was too swollen to measure; the material was not possible to retain in the WRV cup with standard mesh.

**Figure 8:**
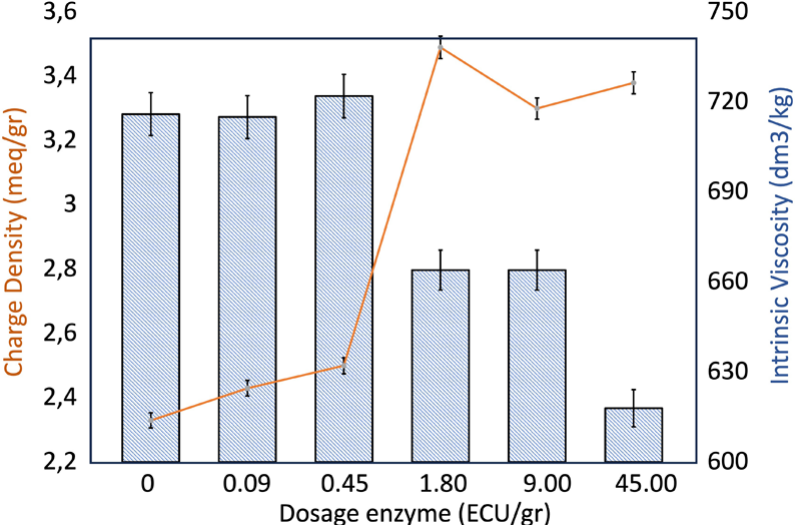
Comparison of intrinsic viscosity and charge density with varying enzyme treatment dosages, 0, 0.09 0.45, 1.80, 9.00, and 45.00 ECU/g.

## 5 Conclusions

This study highlights the importance of combining enzymatic pretreatment with periodate oxidation and Schiff base chemistry for the efficient functionalization of cellulose. While oxidation and cationization have previously been studied in combination, integrating them with enzymatic activation in a three-step sequence demonstrated a synergistic effect that significantly enhances the functional performance of the final product.

The observed improvement is likely due to more than just increased reactivity or a reduced degree of polymerization. Instead, it appears to involve fiber wall loosening and enhanced swelling, triggered by chain cleavage in the less ordered (amorphous) regions of cellulose.^12,29^ These structural modifications increase hydroxyl group accessibility, enabling more effective periodate oxidation and, consequently, the introduction of additional aldehyde groups. These aldehyde sites serve as key reactive centers for Schiff base formation in deep eutectic solvents (DES). These results are in line with earlier reported observations that endoglucanases can increase the reactivity of the cellulose in the viscose process,^23^ lower energy consumption during nanocellulose production by homogenization,^4,24^ and increase the cellulose solubility in alkaline media.^12^

These findings not only clarify the synergistic effect of enzyme-assisted modification but also highlight the relevance of this pathway for industrial applications. The resulting CDAC materials show potential as strength additives and additives for flocculation and coating systems. Moreover, in accordance with the principles of green chemistry,^1^ pretreatment of pulps with endoglucanase, as described in this study, could enhance fiber reactivity, thus leading to lower chemical consumption and more sustainable processes, presenting an interesting possibility for commercial production.
